# Metabolomics Approach Reveals Integrated Metabolic Network Associated with Serotonin Deficiency

**DOI:** 10.1038/srep11864

**Published:** 2015-07-08

**Authors:** Rui Weng, Sensen Shen, Yonglu Tian, Casey Burton, Xinyuan Xu, Yi Liu, Cuilan Chang, Yu Bai, Huwei Liu

**Affiliations:** 1 Beijing National Laboratory for Molecular Sciences, Key Laboratory of Bioorganic Chemistry and Molecular Engineering of Ministry of Education, Institute of Analytical Chemistry, College of Chemistry and Molecular Engineering, Peking University, Beijing 100871, China; 2 Laboratory Animal Centre, Peking University, Beijing 100871, China; 3 Department of Chemistry and Center for Biomedical Science & Engineering, Missouri University of Science and Technology, Rolla, MO 65409, USA

## Abstract

Serotonin is an important neurotransmitter that broadly participates in various biological processes. While serotonin deficiency has been associated with multiple pathological conditions such as depression, schizophrenia, Alzheimer’s disease and Parkinson’s disease, the serotonin-dependent mechanisms remain poorly understood. This study therefore aimed to identify novel biomarkers and metabolic pathways perturbed by serotonin deficiency using metabolomics approach in order to gain new metabolic insights into the serotonin deficiency-related molecular mechanisms. Serotonin deficiency was achieved through pharmacological inhibition of tryptophan hydroxylase (Tph) using p-chlorophenylalanine (pCPA) or genetic knockout of the neuronal specific Tph2 isoform. This dual approach improved specificity for the serotonin deficiency-associated biomarkers while minimizing nonspecific effects of pCPA treatment or Tph2 knockout (Tph2-/-). Non-targeted metabolic profiling and a targeted pCPA dose-response study identified 21 biomarkers in the pCPA-treated mice while 17 metabolites in the Tph2-/- mice were found to be significantly altered compared with the control mice. These newly identified biomarkers were associated with amino acid, energy, purine, lipid and gut microflora metabolisms. Oxidative stress was also found to be significantly increased in the serotonin deficient mice. These new biomarkers and the overall metabolic pathways may provide new understanding for the serotonin deficiency-associated mechanisms under multiple pathological states.

Serotonin is an important neurotransmitter that broadly functions in the regulation of multiple physiological systems, including the cardiovascular, pulmonary, gastrointestinal, genitourinary systems and the central nervous system (CNS)^1^. It participates in the modulation of various neurophysiological processes such as pain perception, energy balance, appetite, sleep, circadian rhythm and aging; neuropsychological processes such as perception, mood, learning, memory, stress and addiction; and behaviors such as play, social perception, aggression, cooperation, mating and sexuality^1^,^2^,^3^.

Efforts to understand serotonin functionality and signaling mechanisms have primarily focused on its 12 heterotrimeric guanine nucleotide binding protein-coupled receptors and one additional ligand-gated ion channel which have been grouped into seven distinct classes (5-HT_1_ to 5-HT_7_)^4^. This conventional approach to understand serotonin’s function in disease is reflected by the numerous drugs that have been developed to target serotonin receptors^5^,^6^,^7^. However, considerable challenges have emerged related to the non-specificity of serotonin receptors to individual biological processes. Consequently, many serotonergic drugs have wide-ranging side effects^1^. One such example is behavioral aggression which is regulated by 5-HT_1A_, 5-HT_1B_ and 5-HT_2A_ receptors^8^,^9^,^10^; however, the 5-HT_1B_ receptor modulates not only aggression but also migraine, locomotion, drug abuse reinforcement, depression and anxiety^11^. These complicated, non-specific receptor-phenotype relationships represent a significant shortcoming in receptor-based approaches to understand serotonin pathophysiology. New approaches are therefore urgently needed to better understand serotonin mechanisms and manage the remarkably large number of pathological conditions.

Metabolomics, the downstream product of genomics, transcriptomics and proteomics, is an emerging ‘-omics’ approach of system biology that has provided often unexpected and unique insights into various biological processes. Unlike the genome or proteome, changes in the metabolome are rapid and represent the final response of an organism to both internal and external stimuli. Hence, metabolomics is particularly conducive to identifying pathophysiologically affected processes and moreover elucidating novel physiological and pathological mechanisms^12^. While metabolomics has been previously applied to several serotonin-related diseases, including depression^13^, schizophrenia^14^ and Parkinson’s disease^15^, its application to serotonin deficiency has not yet been explored and described.

This study therefore sought to identify novel serum metabolites that were significantly altered in serotonin deficient mice compared with control mice. Serotonin deficiency was achieved through two orthogonal routes which included (1) use of p-chlorophenylalanine (pCPA) to pharmacologically inhibit tryptophan hydroxylase (Tph), a rate-limiting enzyme in serotonin biosynthesis^16^,^17^ and (2) genetic knockout of the Tph2 isoform. This dual approach provided specificity for the serotonin deficiency-associated biomarkers from nonspecific effects of either pCPA treatment or Tph2 knockout (Tph2-/-). Ultra-performance liquid chromatography - mass spectrometry was used to pilot novel biomarkers and metabolic pathways in both non-targeted and targeted metabolomics manners and elucidate the fine serotonin deficiency-associated molecular mechanisms.

## Results

### Non-targeted metabolic profiling of pCPA-treated mice

**Figure 1:**
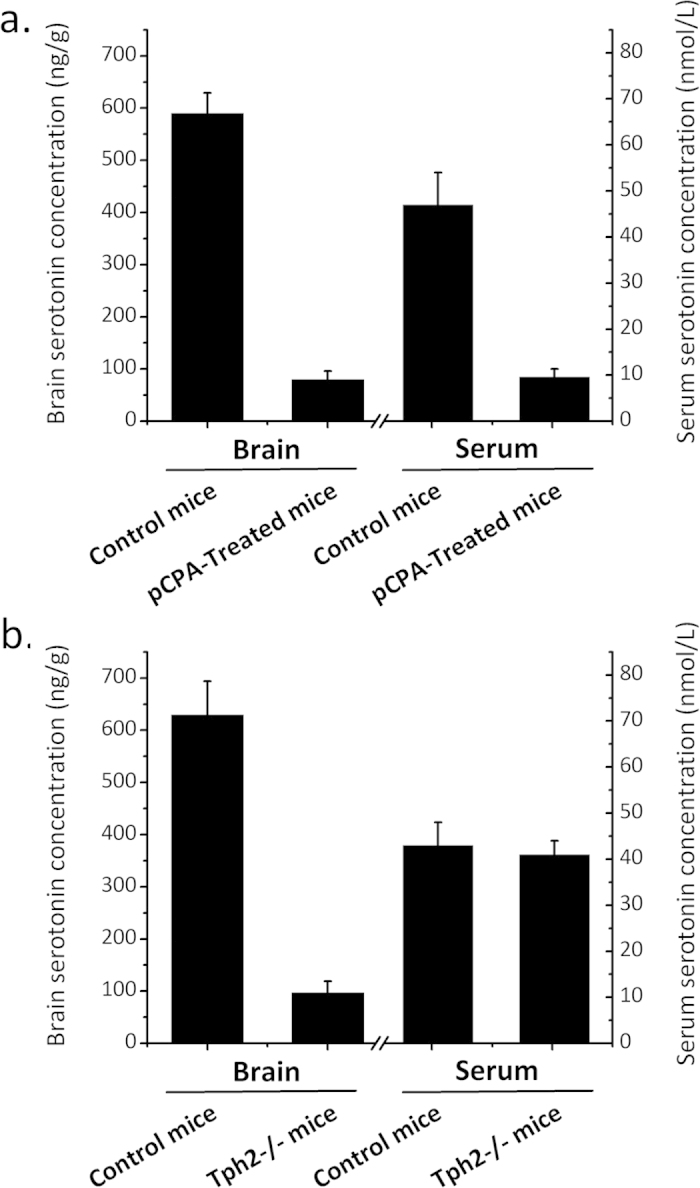
Brain and serum serotonin concentrations of the a) pCPA-treated mice (n = 10) and b) Tph2-/- mice (n = 10). Data are expressed as mean values ± standard deviation.

**Figure 2:**
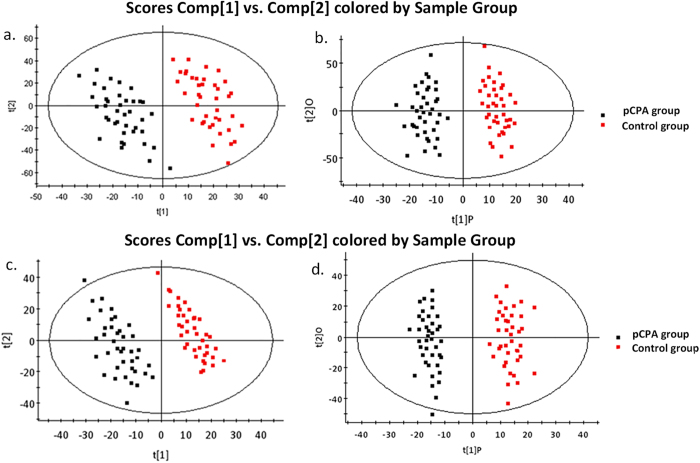
Multivariate statistical analysis results of serum metabolites in the pCPA-treated mice (n = 40) and the control mice (n = 40). **a**) PCA scores plot (R^2^X = 0.447, Q^2^ = 0.77) and **b**) OPLS-DA scores plot (R^2^X = 0.416, R^2^Y = 0.713, Q^2^ = 0.57) in ESI+ mode, ^c^) PCA scores plot (R^2^X = 0.455, Q^2^ = 0.82) and **d**) OPLS-DA scores plot (R^2^X = 0.417, R^2^Y = 0.498, Q^2^ = 0.66) in ESI- mode.

Mice were treated with 500 mg/kg pCPA or saline for three consecutive days. Serotonin levels were significantly reduced in both the brains and sera of the pCPA-treated mice compared with control mice (Fig. 1a). Non-targeted metabolomics approach using ultra-performance liquid chromatography - quadrupole time-of-flight mass spectrometry (UPLC-QTof-MS) was employed to identify potential biomarkers perturbed by the pharmacologically induced serotonin deficiency in the pCPA-treated mice. Principal component analysis (PCA) was performed to highlight key differences between the pCPA-treated and control mice (Fig. 2a,c). Orthogonal partial least squares discriminant analysis (OPLS-DA) further demonstrated a clear metabolic differentiation between the two sample groups (Fig. 2b,d). Variable importance for projection (VIP) scores were obtained for each metabolite based on its individual contribution to the statistical discrimination. In total, 33 preliminary metabolites were found to meet the selection criteria (VIP > 2.0, *p-*value < 0.05, fold change > 1.5) and selected for further characterization.

Several unidentified metabolites also met the selection criteria and have been outlined in Supplementary Table S1 which included retention times, precursor ions (m/z), fold changes, *p*-values and VIP scores. Limited by the current metabolomics identification techniques, they haven’t been identified. But we do believe they are valuable and helpful for relevant researchers. We will keep working on them in further research.

### Targeted pCPA dose-response study

**Figure 3:**
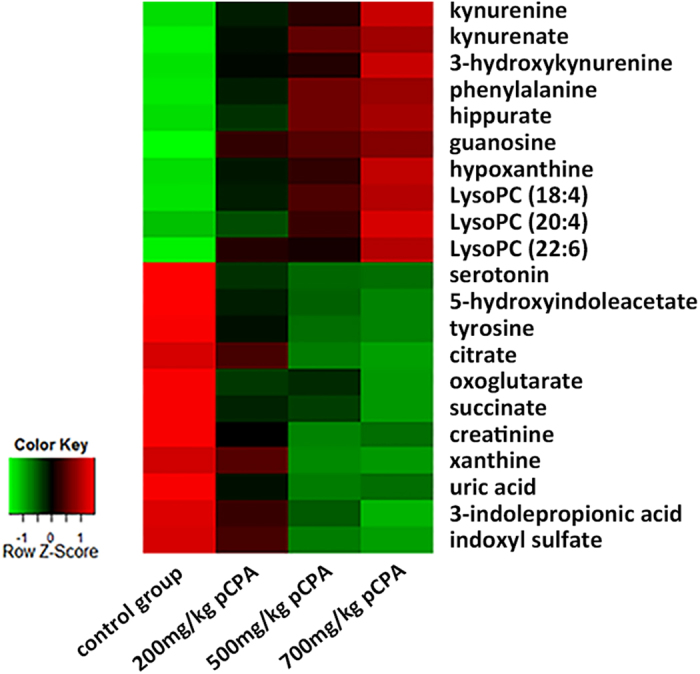
Heat map denoting fold changes (over normalized means) of the 21 biomarkers in mice injected with increasing dosages of pCPA and the control mice (n = 10). Columns correspond to different mice groups, and rows correspond to the altered metabolites. Shades of red represent elevated levels of metabolite, and shades of green represent reduced levels of the metabolites.

A pCPA dose-response study was conducted to confirm the relationships between the 33 preliminary identified metabolites and the pCPA-induced serotonin deficiency. Four groups of mice were treated with variable pCPA doses and the resulting candidate biomarker levels were semi-quantitatively compared. The 33 candidate biomarkers were analyzed using a triple quadrupole mass spectrometer (QQQ-MS) operating under multiple reaction monitoring (MRM) mode. Twenty-one of these candidates were found to be affected by the pCPA treatment dose (Supplementary Figure S1). The 21 biomarkers were therefore considered to be directly correlated with serotonin deficiency and selected as the reliable biomarkers of serotonin deficiency. Specifically, up-regulated metabolites included kynurenine, kynurenate, 3-hydroxykynurenine, phenylalanine, hippurate, guanosine, hypoxanthine and lysoPCs, while down-regulated metabolites included serotonin, 5-hydroxyindoleacetate, tyrosine, xanthine, uric acid, citrate, oxoglutarate, succinate, creatinine, 3-indolepropionic acid and indoxyl sulfate (Fig. 3). Statistics for each compound have been summarized in Table 1.

### pCPA-induced pathway analysis

For the 21 biomarkers found to be associated with pCPA-induced serotonin deficiency, a metabolic pathway analysis was conducted using MetaboAnalyst 2.0. Using this approach, 13 specific metabolic pathways were found to be perturbed (Supplementary Figure S2, Supplementary Table S2). The individual contribution from each perturbed pathway was visualized by plotting the log *p*-value from the pathway enrichment analysis against the pathway impact valued obtained from the pathway topological analysis. Among them, the most relevant and important ones were phenylalanine, tyrosine and tryptophan biosynthesis, phenylalanine metabolism, tryptophan metabolism, citric acid cycle and purine metabolism. In addition to the Kyoto Encyclopedia of Genes and Genomes (KEGG) database-based pathway correlation results, changes in the citric acid cycle and serum creatinine may suggest possible disruption of energy metabolism. The up-regulated lysoPCs indicated the perturbation of the lipid metabolism while the changes of the gut microflora products indicated the gut microflora perturbation (Supplementary Table S3).

### Non-targeted metabolic profiling of Tph2-/- mice

**Figure 4:**
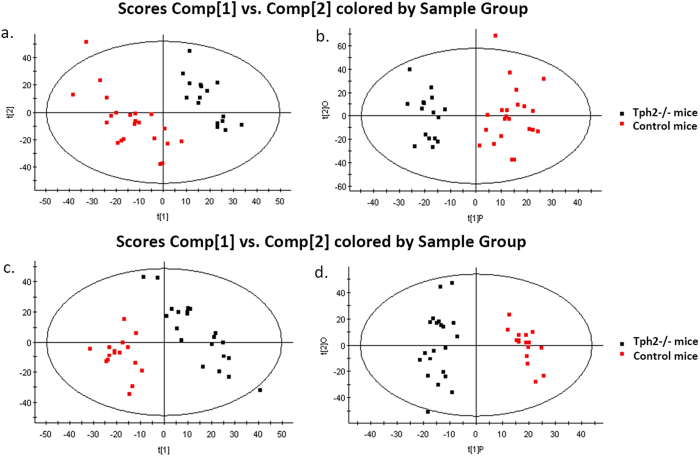
Multivariate statistical analysis results of serum metabolites in the Tph2-/- mice (n = 20) and the control mice (n = 20). **a**) PCA scores plot (R^2^X = 0.536, Q^2^ = 0.84) and **b**) OPLS-DA scores plot (R^2^X = 0.518, R^2^Y = 0.453, Q^2^ = 0.61) in ESI+ mode, **c**) PCA scores plot (R^2^X = 0.533, Q^2^ = 0.86) and **d**) OPLS-DA scores plot (R^2^X = 0.493, R^2^Y = 0.467, Q^2^ = 0.69) in ESI− mode.

Non-targeted metabolic profiling using UPLC-QTof-MS was used to identify potential metabolites perturbed by the genetic serotonin deficiency in the Tph2-/- mice. The genetic knockout of the Tph2 isoform caused a significant decrease of brain serotonin levels although the serum serotonin concentrations remained unchanged (Fig. 1b). PCA and OPLS-DA demonstrated clear differences between the Tph2-/- mice and control mice (Fig. 4). A total of 17 identifiable metabolites were found to meet the selection criteria (VIP > 2.0, *p*-value < 0.05, fold change > 1.5). Multiple unidentified candidate biomarkers also met the selection criteria and have been described in Supplementary Table S4. Specifically, up-regulated metabolites included kynurenine, 3-hydroxykynurenine, xanthurenate, phenylalanine, hippurate, guanosine, hypoxanthine and lysoPCs, while down-regulated metabolites included 5-hydroxyindoleacetate, tyrosine, creatine, xanthosine, citrate, pyruvate, succinate and uric acid (Table 2).

### Metabolic pathway analysis of Tph2-/- mice

Metabolic pathway analysis of the biomarkers of Tph2-/- mice identified 18 perturbed metabolic pathways (Supplementary Figure S3, Supplementary Table S5). Among them, the most relevant and important ones were phenylalanine, tyrosine and tryptophan biosynthesis, phenylalanine metabolism, citrate cycle, purine metabolism and tryptophan metabolism, which have also been perturbed in the pCPA-treated mice. Changes in the citric acid cycle and serum creatine also suggest possible disruption of energy metabolism while the up-regulated lysoPCs indicated the perturbation of the lipid metabolism (Supplementary Table S6).

### Metabolic pathway network construction

**Figure 5:**
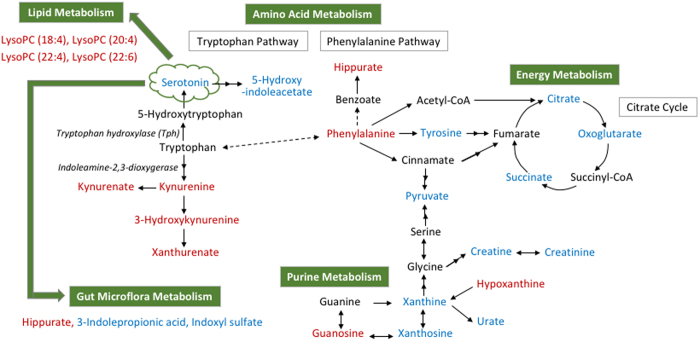
An overview of the integrated metabolic pathway network in response to serotonin deficiency. Red-labeled metabolites indicate up-regulation in mice with serotonin deficiency, while blue-labeled metabolites indicate the down-regulation compared with the control mice. Metabolite relationships were derived from HMDB and KEGG databases. Solid arrows represent direct metabolic reactions, and dashed arrows represent multiple reactions and indirect connections between two metabolites.

Based on the overall 26 biomarkers identified in the pCPA-treated and Tph2-/- mice, metabolic pathway analysis suggested the perturbation of phenylalanine, tyrosine and tryptophan biosynthesis, phenylalanine metabolism, citrate cycle, tryptophan metabolism and purine metabolism, among others (Supplementary Table S7). Visualized pathway analysis has been summarized in Supplementary Figure S4. Overall, the serotonin deficiency-associated biomarkers and perturbed pathways were integrated and broadly categorized to demonstrate the perturbation of amino acid, energy, purine, lipid, and gut microflora metabolisms (Supplementary Table S8, Fig. 5).

### ROS, MDA, T-AOC, SOD, CAT and GPx activity alterations

Reactive oxygen species (ROS) production and malondialdehyde (MDA) levels were significantly increased in both the pCPA-treated mice and Tph2-/- mice compared with control mice (*p*-value < 0.05) (Table 3). To further characterize the antioxidant status in the serotonin deficient mice, the total antioxidant capacity (T-AOC) levels were measured and found to be decreased (*p*-value < 0.05) (Table 3). A decrease in the activities of all measured antioxidant enzymes, including superoxide dismutase (SOD), catalase (CAT) and glutathione peroxidase (GPx) was also obseved (*p*-value < 0.05) (Table 3).

## Discussion

The wide-ranging importance of serotonin to physiological, neuropsychological and behavioral processes underscores the critical need to understand the molecular mechanisms of serotonin deficiency. Indeed, primates with low serotonergic activity were reported to exhibit behaviors indicative of impaired impulse control, unrestrained aggression, social isolation and low social dominance^18^. Serotonin deficiency is also seen in a wide range of disease, including depression^19^,^20^, Alzheimer’s disease^21^,^22^, Parkinson’s disease^23^,^24^ and schizophrenia^12^,^25^, among others. The current study therefore sought to identify novel biomarkers associated with serotonin deficiency to better understand how serotonin deficiency may broadly impact various metabolic pathways under pathological conditions.

Serotonin deficiency was achieved either by the pharmacological inhibition of Tph or genetic knockout of the Tph2 isoform. Notably, Tph has two isoforms that exhibit non-overlapping distribution pattern^26^. Tph1 is expressed in the periphery and pineal gland while Tph2 is expressed exclusively in the CNS^19^. Hence the pCPA treatment would inhibit both Tph2 and Tph1 activity thus affecting both the periphery and CNS serotonin levels. Meanwhile, the knockout of Tph2 isoform would specifically affect CNS serotonin synthesis in the Tph2-/- mice. Indeed, the pCPA treatment caused a significant decrease in both the brain and serum serotonin levels while the knockout of Tph2 only caused a significant decrease of brain serotonin levels in the Tph2-/- mice. Perturbed metabolites observed in both mice models would indicate metabolic dysregulation that originated from CNS serotonin deficiency. This dual model approach minimizes the risk of incorrectly assigning statistically significant metabolites as perturbed serotonin deficiency-associated biomarkers that may actually arise from nonspecific effects of either model.

Non-targeted metabolic profiling of pCPA-treated mice revealed 33 preliminary biomarkers of serotonin deficiency following rigorous multivariate statistical data analyses. By means of a quantitatively targeted pCPA dose-response study, 21 of the above preliminary biomarkers were confirmed having response to pCPA dose, indicating a causative relationship between the 21 biomarkers and serotonin deficiency. Metabolic pathway analysis suggested that these metabolites were associated with 13 specific pathways and most notably the phenylalanine, tyrosine and tryptophan biosynthesis, phenylalanine metabolism, tryptophan metabolism, citric acid cycle and purine metabolism. Pathway correlation also suggested disruption of energy metabolism, lipid metabolism and gut microflora metabolism. Furthermore, the non-targeted metabolomics of the Tph2-/- mice yielded 17 dysregulated metabolites which similarly corresponded with the phenylalanine, tyrosine and tryptophan biosynthesis, phenylalanine metabolism, tryptophan metabolism, citric acid cycle, purine metabolism as well as energy metabolism and lipid metabolism. In total, 26 unique metabolites were found altered in the pCPA-treated and Tph2-/- serotonin deficient mice. These newly identified biomarkers and their associated metabolic pathways constructed the novel serotonin deficiency-affected metabolic pathway network that described the metabolic implications of serotonin deficiency. The metabolic pathway network has been summarized in Fig. 5.

Briefly, tryptophan is an essential amino acid which is metabolized by the serotonin and kynurenine pathways. Importantly, serotonin biosynthesis involves the hydroxylation of tryptophan to 5-hydroxytryptophan (5-HTP) via Tph followed by decarboxylation of 5-HTP to serotonin via aromatic L-amino acid decarboxylase. The Tph-mediated hydroxylation reaction is the rate-limiting step of the serotonin biosynthesis process. Serotonin is then further metabolized to 5-hydroxyindoleacetate and melatonin, among others. In this study where Tph was either pharmacologically inhibited or genetically lacking, serotonin levels significantly decreased. This in turn led to the decrease of 5-hydroxyindoleacetate, one of the downstream serotonin metabolites.

In the kynurenine pathway, kynurenine is synthesized from tryptophan via indoleamine 2, 3-dioxygenase and further metabolized to kynurenate, 3-hydroxykynurenine, 3-hydroxykynurenamine, xanthurenate and others^27^. The elevated levels of kynurenine, kynurenate, 3-hydroxykynurenine and xanthurenate observed in this study indicated activation of the kynurenine pathway under serotonin deficient conditions. This observation may be the result of two competitive pathways for tryptophan where inhibition of one causes the other to experience an apparent activation. Indeed, enhanced indoleamine 2, 3-dioxygenase activity have been observed in patients with depression and anxiety^28^,^29^. Similarly, increased kynurenine levels have been reported in highly depressed patients^30^, while elevated kynurenate levels were found in the cerebrospinal fluid^31^ and cortical^32^ of patients with schizophrenia. In addition, the downstream metabolite 3-hydroxykynurenine has also been widely reported to be increased in multiple serotonin deficiency-related neurodegenerative disorders including Alzheimer’s disease, Parkinson’s disease and Huntington’s disease^33^.

The increased levels of phenylalanine and hippuric acid coupled with decreased tyrosine concentrations observed in this study indicated perturbed phenylalanine metabolism. Specifically, tyrosine is synthesized from phenylalanine via phenylalanine hydroxylase. The simultaneous increase in phenylalanine and decrease in tyrosine suggested decreased phenylalanine hydroxylase activity. This conclusion appeared to agree with previously reported elevation of plasma phenylalanine-tyrosine ratios in depressed patients^34^ as well as decreased levels of tetrahydrobiopterin, which is an essential cofactor of phenylalanine hydroxylase, in patients with depression^35^, schizophrenia and schizoaffective disorder^36^.

Decreased levels of citrate, oxoglutarate, succinate and pyruvate in serotonin deficient mice suggested deactivation of the citrate cycle, which represents a critical energy metabolism pathway that involves the oxidation of carbohydrates, fats and proteins. Notably, the citrate cycle occurs in the mitochondrial matrix, which is particularly sensitive to free radical damage based on its role as the primary source for intracellular free radicals^37^. Our oxidative stress data indicated a significantly 70% ROS increase as well as a 40%–60% MDA increase in the pCPA-treated and Tph2-/- mice. Considerably decreased antioxidant capacities measured from T-AOC, SOD, CAT and GPx assays were also noted. These results implied that serotonin deficiency was related to systemically increased oxidative stress that may extend to deregulated citrate cycle metabolism. Increased cellular oxidative stress and mitochondrial dysfunctions have been similarly reported in several diseases associated with serotonin deficiency, including schizophrenia^38^, Parkinson’s disease and Alzheimer’s disease^39^. Decreased levels of creatine and creatinine in the serotonin deficient mice may also indicate altered energy metabolism. Specifically, these two compounds are the downstream products of creatine phosphate, an important cellular energy carrier. These findings were in accordance with previous reports that serotonin is an important energy regulator within the body^40^,^41^. Serotonin deficiency induced energy metabolism imbalances were also observed in depression^42^.

Purine metabolism deactivation was noted by decreased xanthine, xanthosine and uric acid concentrations coupled with increased guanosine and hypoxanthine levels. Given that purine metabolism is a major metabolism of a homeostatic response of mitochondria to oxidative stress, the disturbance of the mitochondria may lead to the perturbation of purine metabolism, which is also reported in schizophrenia^43^. Specifically, uric acid is an end product of purine metabolism, mainly synthesized from adenine- and guanine-based purines by the enzyme xanthine oxidase. Low levels of uric acid have been associated with a wide range of diseases including depression^44^, schizophrenia^45^, Alzheimer’s disease^46^ and Parkinson’s disease^15^,^47^,^48^.

In addition, increased levels of lysoPC (18:4), lysoPC (20:4), lysoPC (22:4) and lysoPC (22:6) were observed in mice with serotonin deficiency. Lysophosphatidylcholines (LysoPCs) are the products of phosphatidylcholines (PCs) via enzymatic action of phospholipase A_2_ (PLA_2_). Under oxidative stress, lipid peroxidation occurs, evidenced by increased MDA levels observed in both pCPA-treated mice and Tph2-/- mice. As a response, the PLA_2_ activity is enhanced, giving rise to the increase of the lysoPC levels^49^.

Finally, the gut microflora in mice treated with pCPA were found to be perturbed, evidenced by an increase in hippuric acid and a decrease in 3-indolepropionic acid and indoxyl sulfate. Hippuric acid is metabolized from benzoic acid, which is metabolized from the dietary polyphenol 3-hydroxyphenyl propionic acid by the gut microflora^50^. Because the mice were provided a constant diet, changes to hippuric acid were likely the result of gut microbiome perturbation. 3-Indolepropionic and indoxyl sulfate are both metabolites of tryptophan by gut microflora. While peripheral serotonin is an important gastrointestinal signaling molecule that specifically functions as a sensory transducer and a paracrine messenger in the gut^5^, the perturbed gut microflora metabolism may be the result of the peripheral serotonin deficiency in the pCPA-treated mice.

In summary, we used both the non-targeted and targeted metabolomic approach to identify novel biomarkers perturbed by serotonin deficiency and to elucidate the affected metabolic pathways. Serotonin deficiency was achieved either by the pharmacological inhibition of Tph or genetic knockout of the Tph2 isoform. A total of 26 unique metabolites were observed to be dysregulated in the serotonin deficient mice. These findings indicated serotonin deficiency affected tryptophan metabolism, phenylalanine metabolism, energy metabolism, purine metabolism, lipid metabolism and gut microflora metabolism. Further evidence of oxidative stress may provide a mechanistic basis for some of the dysregulated pathways. In-depth study should focus on using these findings as a platform to elucidate specific pathophysiological mechanisms of serotonin deficiency in relation to these metabolic pathway perturbations.

## Methods

### Chemicals and reagents

Formic acid (HPLC grade) was purchased from Dikma Technologies Inc. (Lake Forest, CA, USA). Ultra-pure water was obtained from Hangzhou Wahaha Group Co., Ltd. (Zhejiang, China). Methanol (HPLC grade), acetonitrile (HPLC grade), chloroform (HPLC grade), isopropanol (HPLC grade), Tween, pCPA, serotonin hydrochloride, 5-hydroxyindoleacetate, kynurenine, kynurenate, 3-hydroxykynurenine, phenylalanine, tyrosine, sodium hippurate hydrate, creatinine, guanosine, hypoxanthine, 3-indolepropionic acid, citrate, oxoglutarate, succinate, xanthine, uric acid and indoxyl sulfate potassium salt were purchased from Sigma-Aldrich (St. Louis, MO, USA). T-AOC assay kit with Ferric Reducing Ability of Plasma (FRAP) method, SOD assay kit, total GPx assay Kit and lipid peroxidantion MDA assay kit were purchased from Beyotime Institute of Biotechnology (Jiangsu, China). CAT assay kit and reactive oxygen species assay kit were obtained from Nanjing Jiancheng Bioengineering Institute (Jiangsu, China).

### pCPA treatment

Vertebrate experiments were approved by the institutional review board, the Institutional Animal Care and Use Committee of Peking University and all experiments were carried out in accordance with the approved guidelines and regulations. Animal studies were conducted at a AAA-certified animal facility at the Laboratory Animal Center of Peking University (LAC-PKU). Adult C57BL/6 J male mice between 11 and 13 weeks old were acquired from the Vital River Laboratories (Beijing, China) and housed in the LAC-PKU. The mice were housed in a controlled environment at 22–24 °C and a relative humidity of 40%–60%, and supplemented with 12 h light/dark cycles. Food and tap water were given ad libitum. Mice were housed individually for one week prior to the experiment to ensure adequate adjustment time.

The pCPA treatment procedures used in this study have been described elsewhere^51^, but a few minor modifications were made in the present study. Specifically, mice were randomly divided into pCPA and saline control groups, each comprising 40 samples. Mice of the pCPA group were injected intraperitoneally with 500 mg/kg pCPA per day for three consecutive days, while the control group received saline solution instead. In the pCPA dose-response study, a new batch of 40 mice were randomly divided into four groups and injected intraperitoneally with either 1) saline, 2) 200 mg/kg pCPA, 3) 500 mg/kg pCPA or 4) 700 mg/kg pCPA. pCPA was suspended in 1% Tween saline.

### Tph2-/- mice

Tph2-/- mice were provided by Prof. Yi Rao, generated and genotyped as previously described^51^,^52^,^53^. Mice were housed individually in a controlled environment at 22–24 °C and a relative humidity of 40%–60%, and supplemented with 12 h light/dark cycles. Food and tap water were given ad libitum. All mice used were between 12 and 16 weeks old.

### Sample collection

Mice were anesthetized with pentobarbital sodium followed by blood collection from the orbital sinus at 9 am to 10 am. Upon one hour of sitting at room temperature, the blood was centrifuged at 3000 *xg* to extract sera. All serum samples were then immediately stored at −80 °C. After blood collection, mice were euthanized immediately and the brains were obtained on ice.

### Determination of brain and serum serotonin concentrations in pCPA-treated and Tph2-/- mice

Brain and serum serotonin concentrations of both the pCPA-treated mice and Tph2-/- mice were determined by UPLC-QTof-MS. Mice brain were weighed and homogenized in cold methanol (−20 °C) (4 mL methanol per gram of brain tissue) while 100 μL serum samples were mixed with 400 μL cold methanol. The homogenate or the mixture was then centrifuged at 10,000 *xg* for 15 min. The supernatant was dried under nitrogen and resuspended with 150 μL pure water, 120 μL chloroform and 30 μL isopropanol. After centrifugation, the upper aqueous layer was injected into the UPLC-QTof-MS system for analysis. UPLC-QTof-MS analysis was performed under the same conditions used in the non-targeted metabolic profiling. The serotonin concentrations were calculated using the Waters Masslynx software (version 4.1) based on the standard sample.

### UPLC-QTof-MS analysis

Prior to analysis, the serum samples were naturally thawed at 4 °C. A total of 100 μL serum was added into 400 μL methanol (−20 °C) and the mixture was vortexed vigorously to precipitate protein. After centrifugation, the supernatant was transferred to a 1.5 mL auto-sampler vial for analysis.

A quality control (QC) sample was prepared by mixing equal volumes (10 μL) from each serum sample. This “pooled” sample was used to estimate a “mean” profile representing all the analytes encountered during analysis^54^.

UPLC analysis was performed on an ACQUITY UPLC HSS T3 column (100 mm × 2.1 mm, 1.8 μm; Waters Corp., Dublin, Ireland) using an ACQUITY UPLC (Waters Corp., Milford, USA). A 5 μL injection was made onto the column, which was maintained at 30 °C and eluted with A) water (0.1% (v/v) formic acid, 2% (v/v) acetonitrile) and B) acetonitrile (0.1% (v/v) formic acid) at a flow rate of 0.3 mL/min for 17 min. The gradient duration program was as follows: 0–3 min, 0% B; 3–5 min, 0–30% B; 5–12 min, 30–100% B; 12–17 min, 100% B and re-equilibrated with 0% B for 3 min.

MS analysis was conducted using a Xevo QTof mass spectrometer (Waters, Manchester, UK), operating in both positive (ESI +) and negative (ESI–) electrospray ionization modes. The parameters were as follows: mass range, 50–1,000 Da; scan time, 0.3 s; capillary voltage, 3.0 kV (ESI+)/2.6 kV (ESI–); sample cone voltage, 30 V; extraction cone voltage, 4.0 V; source temperature, 120 °C; desolvation temperature, 450 °C; cone gas flow, 40 L/h; desolvation gas flow, 700 L/h. The data were acquired in MS^E^ mode, in which the centroid MS spectra and the auto MS/MS spectra with collision energies ramping from 10–40 eV were acquired simultaneously. Leucine-enkephalin (278.1141 Da, 556.2771 Da in ESI+ mode and 236.1035 Da, 554.2615 Da in ESI− mode) was used as a lock mass standard to ensure real-time accuracy.

The pooled “QC” sample was injected six times at the beginning of the analysis batch to ensure system equilibrium and then every 10 samples to further monitor the analysis stability^55^,^56^. All samples were injected randomly in the batch. Six metabolites with different *m/z* values and polarities were selected for quality control/quality assurance purposes under ESI+ and ESI− modes separately. The metabolite retention times and selected mass to charge ratios have been tabulated in Supplementary Table S9, indicating good system stability and repeatability.

### UPLC-QTof-MS data processing

The data were analyzed using Waters Masslynx and Makerlynx XS software (version 4.1). Raw data were deconvoluted, aligned, normalized and assembled into a data matrix. Data were aligned with a 0.01 Da mass tolerance and a retention time window tolerance of 0.1 min, followed by filtering using the “80% rule”^57^. Then the data matrix was mean-centered, pareto-scaled and analyzed by PCA and OPLS-DA. Metabolites with VIP scores larger than 2 were considered to be statistically significant. In addition to VIP scores, Mann-Whitney *U* tests were used to compare metabolite levels between the pCPA-treated/Tph2-/- mice and control mice using SPSS 20.0. Finally, Fold changes were calculated from the arithmetic mean values of the pCPA-treated/Tph2-/- and control groups.

### UHPLC-QQQ-MS based dose-response study

The pCPA dose-response study was performed using an Agilent 1290 Infinity LC system (Agilent Technologies, Santa Clara, CA) coupled with an Agilent 6460 Triple Quadrupole mass spectrometer (Agilent Technologies, Santa Clara, CA). UHPLC analysis was also performed with the ACQUITY UPLC HSS T3 column (100 mm × 2.1 mm, 1.8 μm; Waters Corp., Dublin, Ireland) at 30 °C. The gradient duration program was the same as that used in the UPLC-QTof-MS analysis.

The ultra-high performance liquid chromatography-triple quadrupole mass spectrometry (UHPLC-QQQ-MS) analysis was performed under MRM mode. The parameters were as follows: dry gas temperature, 350 °C; dry gas flow, 10 L/min; sheath gas temperature, 350 °C; sheath gas flow, 10 L/min, nebulizer pressure, 40 psi; capillary entrance voltage, 4000 V. Optimized precursor/fragment ions and collision energies were as follows, positive ion mode: 114.1 → 86.1, 10 eV; 137.0 → 110.0, 30 eV; 160.1 → 117.1, 40 eV; 166.1 → 120.1, 20 eV; 177.1 → 160.1, 20 eV; 180.1 → 105.0, 10 eV; 182.1 → 123.0, 30 eV; 182.1 → 136.1, 10 eV; 190.1 → 130.1, 40 eV; 190.1 → 144.0, 20 eV; 192.1 → 146.1, 30 eV; 209.1 → 146.1, 20 eV; 225.1 → 110.1, 10 eV; 284.1 → 152.1, 30 eV; 325.0 → 97.0, 30 eV; 364.1 → 152.1, 40 eV; 514.3 → 184.1, 30 eV; 516.3 → 462.3, 20 eV; 542.3 → 184.1, 30 eV; 566.3 → 184.1, 30 eV; negative ion mode: 117.0 → 73.0, 20 eV; 145.0 → 101.0, 10 eV; 151.0 → 108.0, 20 eV; 152.1 → 122.0, 20 eV; 167.0 → 124.0, 10 eV; 180.1 → 119.1, 30 eV; 191.0 → 87.0, 20 eV; 199.2 → 176.6, 30 eV; 199.0 → 91.1, 20 eV; 212.0 → 80.0, 30 eV; 355.1 → 191.1, 20 eV; 391.3 → 283.3, 40 eV; 407.3 → 343.3, 40 eV. Data were analyzed using the MassHunter Workstation Software (version B.06.00).

### Metabolite identification

Selected biomarkers were initially compared with comprehensive online databases (METLIN: http://metlin.scripps.edu/, HMDB: http://www.hmdb.ca/, KEGG: http://www.kegg.com/, LIPIDMAPS: http://www.lipidmaps.org/ and MassBank: http://www.massbank.jp/) using exact *m/z* values and MS/MS fragments. The identification information was then confirmed by chemical standards with the exact *m/z* values, MS/MS fragments and retention times. MS/MS spectra were acquired with collision energies of 10, 20 and 40 eV.

### Metabolic pathway analysis (MetPA) by MetaboAnalyst

Metabolic Pathway Analysis (MetPA) by MetaboAnalyst 2.0 (www.metaboanalyst.ca/) was used to sort the significantly altered metabolites associated with serotonin deficiency into biologically relevant metabolic pathways. MetPA is a web-based application that combines pathway enrichment analysis and network topological analysis, to aid in the visualization of metabolomics data within the biological context of metabolic pathways^58^,^59^. Notably, the MetPA database includes metabolic pathways encompassing 21 model organisms including the mice used in this study.

### Determination of T-AOC, SOD, CAT, GPx, ROS and MDA levels and activities

The experimental procedures for determining the T-AOC, SOD, CAT, GPx, ROS and MDA activities were based on the protocols provided by Beyotime Institute of Biotechnology and Nanjing Jiancheng Bioengineering Institute. The T-AOC levels, MDA levels, SOD, CAT and GPx activities were determined using mice serum while ROS production was measured using brain. T-AOC was measured by reduction of Fe^3+^ -TPTZ complex to the ferrous form Fe^2+^ -TPTZ. SOD activity was determined using the xanthine/xanthine oxidase method. MDA activity was measured by analyzing the reaction of MDA with thiobarbituric acid (TBA), which forms an MDA-TBA adduct that absorbs strongly at 535 nm. Protein content was measured by the Braford method^60^ using bovine serum albumin (BSA) as a standard protein. All data were presented as mean ± standard deviation (SD). Statistical analysis was performed with a one-way analysis of variance (ANOVA) using SPSS 20.0. Results with *p*-values less than 0.05 were considered statistically significant. All experiments were repeated at least three times.

## Additional Information

**How to cite this article**: Weng, R. *et al.* Metabolomics Approach Reveals Integrated Metabolic Network Associated with Serotonin Deficiency. *Sci. Rep.* **5**, 11864; doi: 10.1038/srep11864 (2015).

## Supplementary Material

Supplementary Information

## Figures and Tables

**Table 1:** Metabolites selected as biomarkers of pCPA-induced serotonin deficiency.

Metabolite	HMDB ID	*m/z*	Representative MS/MS fragment ions (*m/z*)^a^	ESI+			ESI−		
				Fold change^b^	*p* value^c^	VIP^d^	Fold change^b^	*p* value^*c*^	VIP^d^
serotonin	HMDB00259	177.1009	160.0756, 132.0812, 115.0540*	−12.2	2.67 × 10^−5^	17.27			
5-hydroxyindoleacetate	HMDB00763	192.0653	146.0595, 119.0484, 118.0656, 91.0566*	−4.5	9.24 × 10^−6^	12.76			
kynurenine	HMDB00684	209.0912	192.0653, 146.0596, 118.0650, 94.0656*	2.8	1.28 × 10^−5^	9.21			
kynurenate	HMDB00715	190.0517	172.0407, 144.0455, 116.0511, 89.0402*	1.8	4.98 × 10^−5^	8.34			
3-hydroxykynurenine	HMDB00732	225.0866	190.0493, 162.0544, 110.0597*	1.8	1.59 × 10^−2^	2.54			
phenylalanine	HMDB00159	166.0855	120.0806, 103.0549, 91.0542, 77.0396*	2.3	6.29 × 10^−6^	19.22			
tyrosine	HMDB00158	182.0812	165.0548, 136.0753, 119.0491, 91.0541*	−1.6	5.22 × 10^−4^	6.52			
hippurate	HMDB00714	180.0650	105.0338, 77.0394, 51.0230*	2.9	2.68 × 10^−4^	7.14	2.2	6.22 × 10^−3^	6.27
creatinine	HMDB00562	114.0687	86.0741, 72.0480*	−3.7	9.22 × 10^−3^	4.87			
guanosine	HMDB00133	284.0983	152.0558, 135.0294, 110.0341*	2.5	7.66 × 10^−3^	6.59			
hypoxanthine	HMDB00157	137.0457	119.0352, 110.0346, 94.0403, 55.0300*	1.7	5.48 × 10^−4^	11.62			
3-indolepropionic acid	HMDB02302	190.0857	130.0650, 103.0540, 77.0390, 55.0183*	−2.1	1.25 × 10^−3^	2.26			
lysoPC (18:4)	HMDB10389	516.3064	184.0730, 125.0001, 104.1067*	2.1	3.64 × 10^−4^	6.77	1.5	4.32 × 10^−5^	5.79
lysoPC (20:4)	HMDB10395/6	544.3387	184.0732, 104.1070, 86.0965*	2.3	2.77 × 10^−4^	5.65	1.9	6.44 × 10^−4^	3.44
lysoPC (22:6)	HMDB10404	568.3357	184.0730, 104.1068, 86.0966*	1.8	6.43 × 10^−5^	3.27	1.7	1.42 × 10^−3^	5.67
citrate	HMDB00094	191.0193	129.0187, 111.0088, 87.0089**				−2.0	5.12 × 10^−5^	7.27
oxoglutarate	HMDB00208	145.0131	101.0235, 73.0293, 57.0347**				−1.7	8.22 × 10^−3^	6.63
succinate	HMDB00254	117.0195	99.0086, 73.0298, 55.0191**				−1.6	1.29 × 10^−2^	6.94
xanthine	HMDB00292	151.0253	108.0199, 80.0251, 65.9988**				−4.8	3.48 × 10^−4^	16.29
uric acid	HMDB00289	167.0203	124.0147, 96.0204, 69.0100**				−6.3	6.22 × 10^−5^	23.64
indoxyl sulfate	HMDB00682	212.0019	132.0451, 104.0502, 80.9653, 79.9576**				−3.3	7.22 × 10^−3^	9.54

^a^Metabolites labeled with * were confirmed by MS/MS in ESI+ mode, while metabolites labeled with ** were confirmed by MS/MS in ESI–mode.

^b^Fold change was calculated from the arithmetic mean values of the pCPA group and the control group. Fold change with a positive value indicates a relatively higher concentration in the pCPA-treated mice, while a negative value indicates a relatively lower concentration compared with the control mice.

^c^*P*-values were determined by the Mann-Whitney *U* test.

^d^VIP denotes variable importance for projection where values larger than 2.00 reflects high contribution to the distinction between the pCPA group and the control group.

**Table 2:** Metabolites selected as biomarkers in Tph2-/- mice.

Metabolite	HMDB ID	*m/z*	Representative MS/MS fragment ions (*m/z*)^a^	ESI+			ESI−		
				Fold change^b^	*p* value^c^	VIP^d^	Fold change^b^	*p* value^c^	VIP^d^
5-hydroxyindoleacetate	HMDB00763	192.0650	146.0594, 119.0482,118.0650, 91.0564*	−4.7	4.29 × 10^−3^	7.29			
kynurenine	HMDB00684	209.0914	146.0599, 118.0650, 94.0655*	2.1	8.36 × 10^−5^	10.23			
3-hydroxykynurenine	HMDB00732	225.0861	208.0591, 190.0493, 162.0544, 110.0597*	2.3	9.37 × 10^−4^	4.12			
xanthurenate	HMDB00881	206.0442	188.0336, 160.0382, 132.0435*	1.9	4.85 × 10^−3^	5.24			
phenylalanine	HMDB00159	166.0852	120.0806, 103.0537, 91.0542, 77.0396*	3.1	3.68 × 10^−4^	11.86			
tyrosine	HMDB00158	182.0811	165.0548, 136.0753, 119.0494, 91.0541*	−2.4	2.41 × 10^−2^	3.29			
hippurate	HMDB00714	180.0650	105.0339, 77.0392, 51.0230*	2.7	2.68 × 10^−3^	7.44	2.5	2.71 × 10^−2^	9.43
creatine	HMDB00064	132.0774	90.0555, 87.0607, 72.0554*	−1.9	6.37 × 10^−4^	2.97			
xanthosine	HMDB00299	285.0834	153.0398, 136.0130, 110.0338*	−2.2	3.11 × 10^−2^	7.49	–		
guanosine	HMDB00133	284.0983	152.0558, 135.0294, 110.0341*	3.1	4.97 × 10^−3^	6.50			
hypoxanthine	HMDB00157	137.0457	119.0351, 110.0348, 94.0403, 55.0297*	2.8	7.24 × 10^−3^	9.21			
lysoPC (20:4)	HMDB10395/6	544.3387	184.0733, 104.1072, 86.0965*	2.7	5.29 × 10^−4^	4.92			
lysoPC (22:4)	HMDB10401	572.3711	184.0730, 104.1071, 86.0964*	1.9	8.95 × 10^−3^	2.38	2.0	3.14 × 10^−2^	4.28
citrate	HMDB00094	191.0193	129.0184, 111.0088, 87.0090**	–			−2.6	5.46 × 10^−3^	8.73
pyruvate	HMDB00243	87.0081	43.0185**	–			−1.7	1.28 × 10^−2^	3.66
succinate	HMDB00254	117.0195	99.0086, 73.0300, 55.0190**	–			−2.4	8.34 × 10^−4^	9.24
uric acid	HMDB00289	167.0203	124.0147, 96.0203, 69.0097**	–			−4.3	6.41 × 10^−3^	19.17

^a^Metabolites labeled with * were confirmed by MS/MS in ESI+ mode, while metabolites labeled with ** were confirmed by MS/MS in ESI− mode.

^b^Fold change was calculated from the arithmetic mean values of the Tph2-/- mice and the control mice. Fold change with a positive value indicates a relatively higher concentration in the Tph2-/- mice, while a negative value indicates a relatively lower concentration compared with the control mice.

^c^*P*-values were determined by the Mann-Whitney *U* test.

^d^VIP denotes variable importance for projection where values larger than 2.00 reflects high contribution to the distinction between the Tph2-/- group and the control group.

**Table 3:** Antioxidant activities in serotonin deficient mice.

Oxidants	pCPA-treated mice		Tph2-/- mice	
	Control group (n = 20)	pCPA-treated group (n = 20)	Control group (n = 20)	Tph2-/- group (n = 20)
ROS (1/mgpr)	17.61 ± 1.99	29.84 ± 3.21*	20.46 ± 2.21	34.62 ± 2.11*
MDA (μmol/L)	2.24 ± 0.44	3.16 ± 0.88*	2.03 ± 0.36	3.38 ± 1.12*
T-AOC (mmol/L FeSO_4_)	1.32 ± 0.14	0.94 ± 0.07*	1.62 ± 0.21	0.72 ± 0.11*
SOD (U/mL)	1.14 ± 0.10	0.72 ± 0.04*	1.44 ± 0.11	0.62 ± 0.07*
CAT (U/mL)	0.16 ± 0.024	0.13 ± 0.0088*	0.14 ± 0.011	0.079 ± 0.0084*
GPx (U/mL)	2.72 ± 0.33	1.82 ± 0.094*	2.46 ± 0.19	1.64 ± 0.19*

ROS, reactive oxygen species; MDA, malondialdehyde; T-AOC, total antioxidant capacity; SOD, superoxide dismutase; CAT, catalase; GPx, glutathione peroxidase.

Data are expressed as mean values ± standard deviation.

^*^p-value < 0.05 compared with control group. *P*-values were determined by the Mann-Whitney *U* test.
