# Supplementary material for: Metabolomics Approach Reveals Integrated Metabolic Network Associated with Serotonin Deficiency
Source: Sci Rep. 2015 Jul 8;5:11864. doi: 10.1038/srep11864 (PMC4495385; doi:10.1038/srep11864)
Supplement: Supplementary Information [file srep11864-s1.pdf]

Metabolomics Approach Reveals Integrated Metabolic Network Associated with Serotonin Deficiency

Rui Weng[1], Sensen Shen[1], Yonglu Tian[2], Casey Burton[3], Xinyuan Xu[1], Yi Liu[1], Cuilan Chang[1], Yu Bai[1,] *, Huwei Liu[1]

1 Beijing National Laboratory for Molecular Sciences, Key Laboratory of Bioorganic Chemistry and Molecular Engineering of Ministry of Education, Institute of Analytical Chemistry, College of Chemistry and Molecular Engineering, Peking University, Beijing 100871, China

2 Laboratory Animal Cente, Peking University, Beijing 100871, China

3 Department of Chemistry and Center for Biomedical Science & Engineering, Missouri University of Science and Technology, Rolla, MO 65409, USA

Corresponding Author

* E-mail: yu.bai@pku.edu.cn. Phone: +86-10-62758198. Fax: +86-10-62751708.

Supplementary Table S1 Unidentified candidate biomarkers of pCPA-induced serotonin deficiency

	Retention time/min	*m/z*	Fold change[a]	*p* value[b]	VIP[c]
ESI+	7.3625	324.0547	6.62	5.47×10^{-3}	5.73
	9.0816	352.0510	4.49	5.86×10^{-3}	5.08
	5.6483	224.0465	4.20	3.75×10^{-2}	1.94
	9.0819	356.0468	3.28	9.57×10^{-4}	1.71
	10.0987	338.9932	3.16	9.26×10^{-3}	2.19
	10.1176	431.0020	2.83	5.72×10^{-4}	1.53
	8.5833	406.0418	2.78	2.94×10^{-2}	1.23
	9.6320	459.3483	2.63	1.86×10^{-2}	1.14
	10.0377	375.2895	2.39	3.75×10^{-3}	1.28
	11.6329	939.6556	2.08	7.65×10^{-4}	2.22
	7.6590	585.2686	2.07	8.95×10^{-5}	1.26
	7.0540	538.2817	1.99	3.65×10^{-3}	1.83
	9.9271	441.3393	1.96	2.86×10^{-2}	1.18
	8.9168	475.3417	1.90	1.95×10^{-2}	1.01
	10.0376	357.2816	1.89	3.65×10^{-3}	2.31
	7.0588	533.3270	1.83	3.84×10^{-4}	1.36
	7.0531	516.3003	1.78	8.41×10^{-4}	1.41
	1.1520	322.1076	1.73	2.84×10^{-3}	2.54
	7.0530	498.2889	1.73	4.85×10^{-4}	1.28
	9.4896	211.1338	1.62	3.85×10^{-3}	1.06
	7.0816	241.1774	1.61	3.05×10^{-2}	1.18
	6.9517	225.1104	1.60	3.94×10^{-2}	1.11
	7.3735	172.0764	1.59	5.97×10^{-3}	1.23
	12.072	90.9787	1.58	6.55×10^{-4}	1.97
	0.9073	271.9588	1.57	4.65×10^{-4}	1.17
	0.9628	657.1317	1.52	2.91×10^{-2}	1.42
	10.6347	239.1537	1.51	5.80×10^{-3}	2.01
	0.9471	342.0714	1.50	3.72×10^{-3}	1.04
	0.7741	155.0788	-1.51	5.98×10^{-3}	1.54
	5.0014	160.0749	-1.53	3.85×10^{-3}	6.55
	0.7742	449.0111	-1.54	2.04×10^{-4}	1.03
	9.9008	303.6434	-1.56	7.84×10^{-4}	1.13
	0.7966	782.7713	-1.59	8.90×10^{-3}	1.00
	5.0018	177.1021	-1.60	1.83×10^{-2}	4.04
	5.0165	221.0939	-1.62	3.80×10^{-3}	1.28
	1.1531	268.1084	-1.65	5.76×10^{-5}	1.81
	8.5907	614.1832	-1.69	3.95×10^{-3}	2.27
	11.287	558.3536	-1.70	7.89×10^{-4}	1.57
	0.7748	516.9981	-1.71	2.37×10^{-3}	1.35
	10.4732	862.5690	-1.74	5.74×10^{-4}	1.41
	16.8164	369.3521	-1.75	6.76×10^{-4}	3.70
	17.303	180.1251	-1.78	4.84×10^{-3}	4.05
	0.9649	291.0721	-1.92	8.74×10^{-5}	3.01

	10.1501	665.2730	-4.46	4.82×10^{-3}	1.22
ESI-	10.0707	788.3504	6.06	7.49×10^{-4}	9.50
	5.6430	283.0094	5.48	2.78×10^{-4}	5.98
	7.0071	264.9855	5.46	3.25×10^{-3}	3.91
	9.0505	350.0340	5.21	4.73×10^{-3}	4.99
	10.0566	428.9893	5.19	4.78×10^{-3}	5.21
	7.0114	125.0152	5.02	6.86×10^{-5}	3.50
	10.0622	587.0374	5.02	6.89×10^{-4}	6.38
	7.995	400.0178	4.88	5.94×10^{-4}	2.66
	10.0642	496.9777	4.50	3.99×10^{-2}	2.41
	10.0622	297.0096	4.16	9.67×10^{-4}	2.27
	9.0560	418.0203	4.12	5.86×10^{-3}	2.44
	10.0698	856.3374	4.08	4.79×10^{-3}	2.40
	10.0701	856.3451	4.03	3.86×10^{-4}	3.04
	7.1100	334.9940	3.87	7.86×10^{-3}	2.75
	10.0636	377.9914	3.70	7.98×10^{-4}	1.99
	7.1110	402.9771	3.68	5.74×10^{-4}	2.12
	9.0610	358.9900	3.53	7.89×10^{-3}	1.67
	10.0757	496.9691	3.40	3.86×10^{-3}	3.42
	10.0755	924.3298	3.27	9.85×10^{-4}	1.68
	10.0619	583.2061	3.21	7.22×10^{-3}	1.56
	10.0604	583.2180	3.19	5.74×10^{-3}	1.84
	10.0684	390.9090	3.06	7.05×10^{-3}	2.26
	10.0813	856.3289	2.79	3.48×10^{-2}	1.26
	6.0866	349.1473	2.61	5.47×10^{-4}	1.55
	9.9851	459.2677	2.50	7.98×10^{-3}	2.29
	10.6368	455.3094	2.46	3.92×10^{-3}	1.56
	10.6303	455.3200	2.36	6.17×10^{-4}	1.30
	7.6856	453.2853	2.12	2.96×10^{-2}	1.80
	8.4908	453.2830	2.10	4.75×10^{-3}	2.70
	8.4937	407.2789	2.06	4.50×10^{-3}	2.36
	7.6859	407.2804	2.04	4.71×10^{-3}	2.97
	9.9829	437.2888	1.98	6.08×10^{-3}	1.17
	8.4924	475.2676	1.93	6.98×10^{-4}	1.38
	0.9604	335.0472	1.76	4.94×10^{-4}	1.88
	9.9820	391.2854	1.74	7.09×10^{-3}	2.00
	8.4933	473.2534	1.71	3.31×10^{-3}	2.07
	0.8116	834.8262	1.69	3.75×10^{-2}	1.07
	10.3096	133.6157	1.67	8.90×10^{-5}	1.29
	5.6416	245.0108	1.63	5.89×10^{-4}	1.41
	7.9947	475.2656	1.63	3.65×10^{-2}	1.35
	10.1929	295.2268	1.59	7.96×10^{-5}	1.25
	13.5919	61.9890	1.56	3.27×10^{-3}	1.25
	10.8274	297.2414	1.56	7.48×10^{-5}	1.66
	6.9512	201.1113	1.55	6.89×10^{-4}	4.05

6.8509	199.1045	1.54	2.16×10^{-2}	2.72
6.8406	199.0946	1.54	4.58×10^{-3}	1.14
10.2796	241.1803	1.53	6.78×10^{-4}	1.75
5.7626	245.0101	1.51	3.05×10^{-3}	1.28
9.8435	977.5776	-1.50	4.78×10^{-3}	1.80
10.3929	526.2981	-1.51	2.83×10^{-3}	1.10
6.8484	154.4972	-1.51	654×10^{-4}	1.16
1.0128	264.9576	-1.51	4.84×10^{-3}	4.47
9.1126	582.2164	-1.53	2.19×10^{-4}	1.27
9.5202	623.2914	-1.53	5.83×10^{-3}	1.10
9.0752	589.1886	-1.57	9.47×10^{-5}	1.41
5.6032	809.2496	-1.66	3.29×10^{-3}	1.34
0.9622	259.0200	-1.73	5.49×10^{-4}	4.28
5.9692	306.9978	-1.77	6.54×10^{-3}	1.27
11.519	616.2779	-1.94	7.69×10^{-3}	1.25
1.1629	391.1207	-2.05	4.28×10^{-3}	2.46

a) Fold change was calculated from the arithmetic mean values of the pCPA group and the control group. Fold change with a positive value indicates a relatively higher concentration in the pCPA-injected mice, while a negative value indicates a relatively lower concentration compared with the control mice.

b) *P*-values were determined by the Mann-Whitney *U* test.

c) VIP denotes variable importance for projection where values larger than 1.00 reflects high contribution to the distinction between the pCPA group and the control group.

Supplementary Table S2 Summary of the pCPA-induced Metabolic Pathway Analysis (MetPA) results

No.	Pathway Name	Hits[a]	*p*[b]	-log(*p*)	Impact[c]
1	Phenylalanine, tyrosine and tryptophan biosynthesis	2	7.0824E-4	7.2527	1.0
2	Phenylalanine metabolism	2	0.0061988	5.0834	0.40741
3	Tryptophan metabolism	3	0.009048	4.7052	0.19371
4	Citrate cycle (TCA cycle)	3	0.0011993	6.726	0.14721
5	Purine metabolism	4	0.0057049	5.1664	0.06726
6	Alanine, aspartate and glutamate metabolism	2	0.028554	3.556	0.06329
7	Butanoate metabolism	2	0.024215	3.7208	0.0
8	Ubiquinone and other terpenoid-quinone biosynthesis	1	0.033517	3.3957	0.0
9	D-Glutamine and D-glutamate metabolism	1	0.055273	2.8955	0.0
10	Glyoxylate and dicarboxylate metabolism	1	0.18588	1.6827	0.25806
11	Tyrosine metabolism	1	0.39796	0.9214	0.14045
12	Aminoacyl-tRNA biosynthesis	2	0.18107	1.7089	0.0
13	Propanoate metabolism	1	0.2044	1.5877	0.0

a) The ‘Hits’ is the number of matched compound from the uploaded data in the pathway.

b) The ‘*p*’ is the original *p* value calculated from the pathway enrichment analysis.

c) The ‘Impact’ is the pathway impact value calculated from the pathway topological analysis.

Supplementary Table S3 Perturbed metabolic pathways and associated metabolites of pCPA-induced serotonin deficiency

Pathway category	Pathway[a]	Associated metabolites[b]
Amino acid metabolism	Phenylalanine, tyrosine and tryptophan biosynthesis	Phenylalanine↑, Tyrosine↓
	Phenylalanine metabolism	Phenylalanine↑, Hippurate↑, Tyrosine↓
	Tryptophan metabolism	Serotonin↓, 5-Hydroxyindoleacetate↓, Kynurenine↑, Kynurenate↑, 3-Hydroxykynurenine↑
Energy metabolism	Citrate cycle	Citrate↓, Oxoglutarate↓, Succinate↓
	-	Creatinine↓
Nucleotide metabolism	Purine metabolism	Xanthine↓, Uric acid↓, Guanosine↑, Hypoxanthine↑
Lipid metabolism	-	LysoPC (18:4) ↑, LysoPC (20:4) ↑, LysoPC (22:6) ↑
Gut microflora metabolism	-	Hippurate↑, 3-Indolepropionic acid↓, Indoxyl sulfate↓

Notes: a) the column 'Pathway' refers to the KEGG database-based pathway correlation results by MetPA. b) '↑' indicates increased metabolite level of the pCPA-injected mice, while '↓' indicates decreased metabolite level compared with the control mice.

Supplementary Table S4 Unidentified candidate biomarkers in Tph2-/- mice

	Retention time	m/z	Fold change[a]	*p* value[b]	VIP[c]
ESI+	9.6022	987.6415	6.23	2.59×10^{-2}	2.59
	6.6862	496.2730	6.01	3.75×10^{-2}	1.61
	13.5353	826.5392	5.65	7.25×10^{-3}	2.91
	13.6442	443.2080	4.69	7.38×10^{-3}	1.94
	9.5713	987.6500	4.35	4.86×10^{-4}	3.04
	9.5257	452.2688	3.61	4.76×10^{-4}	3.62
	12.7179	804.5511	3.13	2.41×10^{-3}	6.24
	13.6508	828.5532	2.95	9.43×10^{-5}	13.52
	0.7324	118.9430	2.83	4.72×10^{-4}	1.12
	5.6126	515.2682	2.83	5.25×10^{-3}	1.30
	12.9662	732.5593	2.68	4.21×10^{-3}	2.16
	12.7481	354.1834	2.61	4.86×10^{-3}	1.05
	5.5941	515.2622	2.55	9.32×10^{-3}	1.30
	12.7402	354.1736	2.50	9.36×10^{-4}	1.35
	9.5243	434.2700	2.43	2.87×10^{-2}	1.05
	12.7344	352.1792	2.33	4.94×10^{-3}	2.04
	5.3954	599.5998	2.23	3.57×10^{-4}	1.56
	5.3969	599.9449	2.21	9.35×10^{-5}	1.58
	9.6080	516.3069	2.17	6.19×10^{-3}	9.87
	10.6554	959.6463	2.15	7.35×10^{-3}	1.44
	13.6485	778.5327	2.10	5.32×10^{-3}	3.91
	12.7347	311.1565	2.09	1.44×10^{-2}	1.10
	1.1689	153.0414	2.08	9.43×10^{-3}	4.13
	12.5516	367.1907	2.03	3.85×10^{-3}	1.29
	9.5189	452.2773	2.03	4.64×10^{-3}	3.38
	9.3913	516.3074	1.99	3.65×10^{-4}	4.62
	13.6458	804.5510	1.99	9.59×10^{-3}	6.64
	12.0921	723.5406	1.96	8.37×10^{-3}	2.97
	17.0952	328.2720	1.95	9.23×10^{-5}	1.42
	9.3944	494.3234	1.91	5.93×10^{-3}	6.96
	12.2271	90.5080	1.91	7.68×10^{-4}	1.64
	10.8388	510.3548	1.84	4.85×10^{-4}	7.75
	13.6395	350.2677	1.82	5.03×10^{-4}	1.84
	9.5168	588.3065	1.81	8.29×10^{-3}	1.31
	8.1758	342.2663	1.81	4.49×10^{-3}	1.16
	9.5187	474.2621	1.81	5.83×10^{-3}	1.52
	12.2322	725.5593	1.80	8.77×10^{-5}	2.56
	9.5975	494.3226	1.79	4.95×10^{-4}	24.10
	13.835	102.0904	1.76	9.43×10^{-3}	3.63
	13.6585	834.6083	1.75	5.39×10^{-3}	1.87
	13.535	804.5508	1.75	8.87×10^{-4}	8.13
	9.5824	652.2878	1.72	3.87×10^{-3}	1.23
	13.6500	808.5830	1.71	3.95×10^{-3}	2.43

9.8100	568.3407	1.71	3.85×10^{-3}	8.89
10.3142	568.3445	1.70	6.69×10^{-3}	2.26
16.4063	772.6218	1.70	5.72×10^{-3}	1.05
13.6569	848.5493	1.68	8.50×10^{-4}	2.26
9.9556	502.3021	1.67	4.65×10^{-3}	11.07
12.9361	778.5330	1.66	3.96×10^{-3}	3.20
13.6510	756.5569	1.65	3.28×10^{-2}	1.46
9.3827	564.2994	1.64	6.59×10^{-4}	1.56
10.3365	546.3559	1.63	9.95×10^{-5}	10.20
12.9455	828.5516	1.63	3.75×10^{-4}	9.25
9.5845	532.2808	1.59	3.72×10^{-4}	1.90
13.5318	778.5340	1.58	5.48×10^{-3}	3.79
9.5730	104.1072	1.57	4.83×10^{-3}	3.95
13.5286	90.9778	1.57	3.98×10^{-3}	1.24
12.2767	538.3900	1.56	8.63×10^{-3}	2.49
9.5829	601.2890	1.56	4.83×10^{-4}	1.68
9.4252	580.2830	1.54	9.45×10^{-5}	1.12
12.5074	326.3070	1.52	3.84×10^{-4}	1.17
12.4934	512.4890	-1.50	2.98×10^{-4}	1.56
5.9508	225.1103	-1.50	6.77×10^{-3}	1.26
1.0088	61.0115	-1.50	5.74×10^{-3}	1.42
13.7856	432.2775	-1.52	4.86×10^{-3}	1.04
1.0120	102.0548	-1.54	3.85×10^{-3}	2.61
1.1737	165.0533	-1.56	9.89×10^{-3}	9.52
0.9268	132.0760	-1.56	2.73×10^{-2}	11.48
1.2063	218.1387	-1.57	2.85×10^{-3}	3.98
5.3918	244.1218	-1.59	9.82×10^{-4}	1.04
7.0680	281.1745	-1.64	9.52×10^{-3}	1.46
11.8643	282.2792	-1.64	2.75×10^{-4}	1.64
11.4396	456.4085	-1.67	3.29×10^{-4}	1.54
12.3221	139.9894	-1.68	7.73×10^{-3}	2.66
7.0866	223.1677	-1.71	3.95×10^{-3}	1.15
5.7468	334.1408	-1.72	8.49×10^{-5}	1.64
5.5464	144.0672	-1.73	3.62×10^{-4}	1.28
7.9428	300.1990	-1.74	4.86×10^{-4}	1.51
4.9974	297.1444	-1.81	8.57×10^{-4}	1.23
5.6020	132.0451	-1.82	5.72×10^{-3}	1.43
15.5793	342.3357	-1.83	9.06×10^{-3}	1.23
5.5404	218.9702	-1.84	8.40×10^{-3}	1.99
5.1730	173.0811	-1.86	3.75×10^{-3}	1.67
0.9246	304.1603	-1.89	8.76×10^{-4}	1.17
12.3479	758.5732	-1.91	5.87×10^{-3}	3.44
4.9907	297.1511	-1.92	3.95×10^{-4}	1.11
4.9939	182.0796	-1.93	6.59×10^{-4}	1.82
5.9410	176.0379	-1.95	5.83×10^{-3}	1.71

	5.5946	723.8156	-2.10	5.85×10^{-3}	1.02
	5.9448	268.0065	-2.12	2.83×10^{-2}	1.40
	5.9430	246.0242	-2.13	3.97×10^{-3}	1.82
	12.1164	828.5534	-2.16	9.37×10^{-3}	4.26
	8.2415	538.3544	-2.17	9.82×10^{-3}	1.10
	4.9965	311.1243	-2.18	7.93×10^{-4}	2.39
	7.7841	253.1448	-2.18	5.73×10^{-3}	1.43
	5.9416	976.9729	-2.21	3.06×10^{-3}	1.02
	5.9447	134.0272	-2.22	2.85×10^{-2}	2.92
	4.9987	355.1020	-2.27	8.40×10^{-4}	1.06
	12.3282	143.9664	-2.27	3.02×10^{-3}	2.00
	8.4250	395.2067	-2.28	4.86×10^{-3}	1.37
	1.0776	226.0458	-2.44	2.95×10^{-3}	3.62
	12.3292	786.6076	-2.50	8.16×10^{-5}	1.23
	7.8594	512.3348	-2.56	9.57×10^{-5}	5.91
	5.2680	206.0467	-2.57	3.84×10^{-4}	1.03
	7.7835	213.1513	-2.58	7.38×10^{-3}	1.31
	5.5223	390.1849	-2.62	5.62×10^{-3}	1.02
	1.1814	275.1120	-2.63	3.86×10^{-3}	1.05
	11.9498	484.4418	-2.71	8.26×10^{-3}	1.45
	7.8508	534.3170	-2.72	4.86×10^{-4}	3.06
	9.1148	431.2035	-2.74	2.94×10^{-3}	1.53
	0.9382	263.1491	-2.85	3.04×10^{-3}	1.26
	9.1149	211.2071	-2.88	3.86×10^{-3}	1.25
	8.6108	540.3681	-2.95	3.86×10^{-4}	2.35
	9.1160	409.2196	-3.02	9.27×10^{-4}	1.94
	0.9264	164.9270	-3.40	9.46×10^{-4}	2.73
	8.8368	453.3377	-3.69	1.45×10^{-2}	1.36
	0.9373	257.0793	-3.75	5.86×10^{-3}	1.32
	9.1252	211.1975	-3.79	2.83×10^{-4}	1.01
	5.1459	368.0939	-4.33	5.87×10^{-3}	1.13
	8.8351	471.3495	-4.64	3.95×10^{-4}	2.03
ESI-	10.4872	272.4697	2.84	9.75×10^{-3}	1.35
	12.8538	590.3670	2.74	7.72×10^{-4}	4.04
	0.8164	624.5393	2.69	8.64×10^{-5}	1.10
	12.8456	421.1370	2.68	2.93×10^{-3}	1.61
	13.3077	367.1600	2.41	4.76×10^{-4}	2.12
	12.8456	351.1482	2.33	7.82×10^{-3}	3.03
	12.8497	253.2179	2.31	3.76×10^{-3}	3.25
	10.7446	999.6508	2.27	9.27×10^{-3}	1.33
	12.8998	640.3839	2.25	2.84×10^{-2}	1.25
	12.8583	353.1399	2.19	4.86×10^{-4}	12.74
	10.4734	122.8181	2.16	8.36×10^{-3}	1.21
	9.6211	518.2512	2.14	9.90×10^{-3}	1.61
	12.721	655.4700	2.14	8.26×10^{-3}	1.20

9.1904	213.1490	2.11	3.75×10^{-2}	3.26
9.5596	858.2711	2.10	2.31×10^{-2}	1.28
13.7647	135.9694	2.09	4.76×10^{-3}	7.03
13.3038	405.1742	2.06	9.26×10^{-4}	2.76
13.2454	299.2594	2.03	5.99×10^{-4}	3.99
14.8149	265.1477	2.01	3.56×10^{-4}	4.97
9.6961	810.2704	1.99	8.62×10^{-3}	2.12
10.9652	621.3061	1.96	7.50×10^{-3}	1.74
12.6673	327.1259	1.93	2.56×10^{-3}	3.34
1.0011	528.7949	1.93	4.76×10^{-4}	1.05
9.6171	450.2621	1.91	1.83×10^{-3}	3.57
12.8485	321.1982	1.91	9.36×10^{-4}	1.10
12.5163	377.1417	1.87	9.25×10^{-5}	2.44
10.0493	166.2735	1.87	2.65×10^{-4}	1.14
9.6113	522.2631	1.87	2.66×10^{-3}	1.37
12.9998	329.2524	1.84	1.13×10^{-2}	1.59
12.8462	321.2062	1.84	3.04×10^{-3}	1.25
6.9397	155.7055	1.81	4.56×10^{-4}	1.96
9.1914	281.1380	1.81	2.84×10^{-2}	2.07
9.3736	452.2799	1.80	1.93×10^{-3}	1.60
13.7569	366.2269	1.79	5.78×10^{-3}	1.00
10.4744	68.2375	1.79	3.96×10^{-4}	1.38
12.1801	100.9319	1.79	3.97×10^{-4}	2.49
12.1771	271.2276	1.79	9.74×10^{-5}	1.77
10.4746	123.0601	1.75	2.89×10^{-2}	1.16
13.7592	349.2383	1.74	9.67×10^{-5}	1.09
11.1301	297.2418	1.72	8.67×10^{-4}	2.46
0.7545	962.8179	1.71	6.97×10^{-3}	1.47
11.7745	77.2288	1.69	5.59×10^{-3}	1.07
13.0128	429.1731	1.68	7.76×10^{-3}	2.45
9.5449	671.2936	1.67	3.86×10^{-2}	1.26
10.8127	57.1329	1.65	3.65×10^{-2}	1.12
13.9327	407.1875	1.63	9.57×10^{-5}	1.05
12.7183	425.1618	1.63	3.65×10^{-4}	1.26
14.1607	201.8243	1.61	7.72×10^{-3}	1.37
14.8331	409.2066	1.61	5.66×10^{-3}	1.43
10.9290	616.3714	1.60	5.76×10^{-4}	1.48
13.7590	134.8924	1.60	7.53×10^{-4}	4.39
9.4981	538.3145	1.59	9.27×10^{-3}	6.23
13.0800	293.1755	1.58	3.92×10^{-2}	1.58
12.8356	655.4235	1.57	7.75×10^{-3}	4.56
13.7588	646.4296	1.57	3.75×10^{-4}	3.53
11.0153	325.2073	1.56	6.45×10^{-4}	1.20
12.7146	395.2211	1.56	9.34×10^{-4}	2.49
10.2988	590.3462	1.54	7.29×10^{-3}	6.83

9.6275	610.3177	1.54	2.85×10^{-2}	1.58
9.6970	623.2960	1.52	1.94×10^{-2}	1.83
12.7028	427.1574	1.52	6.50×10^{-4}	5.11
13.7629	466.1524	1.51	9.35×10^{-5}	1.32
9.5498	790.2813	1.51	4.65×10^{-4}	1.49
5.3151	257.1007	1.50	7.64×10^{-3}	1.10
7.9429	335.0591	-1.50	3.75×10^{-3}	2.69
4.9430	276.0770	-1.50	2.96×10^{-3}	1.00
1.1223	175.0235	-1.51	8.67×10^{-4}	4.48
1.1079	133.0140	-1.51	9.47×10^{-4}	3.65
8.2303	344.1535	-1.51	3.75×10^{-4}	2.27
5.3666	128.0348	-1.51	6.75×10^{-3}	2.27
6.7888	186.1111	-1.53	5.63×10^{-3}	1.75
4.8856	144.0638	-1.54	6.73×10^{-3}	1.51
0.8701	301.8977	-1.55	9.78×10^{-4}	1.64
5.5167	190.0518	-1.55	3.64×10^{-2}	2.00
1.5234	117.0186	-1.58	5.74×10^{-3}	5.03
5.7436	415.9659	-1.58	4.93×10^{-3}	1.43
5.7463	483.9565	-1.59	5.86×10^{-4}	1.30
6.9987	201.1133	-1.60	3.85×10^{-3}	2.45
5.8259	370.0702	-1.61	7.36×10^{-3}	1.01
7.2624	202.0502	-1.61	5.76×10^{-3}	2.17
6.4011	270.0397	-1.62	9.57×10^{-5}	1.62
4.9520	277.0878	-1.63	3.65×10^{-3}	1.01
6.9953	223.0959	-1.63	8.25×10^{-3}	1.49
11.5081	366.1130	-1.64	4.76×10^{-3}	1.56
5.6907	266.9938	-1.67	7.66×10^{-4}	1.92
5.6969	332.1225	-1.70	3.28×10^{-2}	1.15
0.9289	247.9740	-1.70	3.75×10^{-3}	1.20
0.8554	166.8485	-1.71	8.56×10^{-4}	1.38
6.0720	349.1485	-1.71	5.73×10^{-5}	1.26
1.5029	229.0306	-1.74	4.76×10^{-4}	3.39
1.1564	285.0612	-1.75	9.27×10^{-3}	1.19
5.5186	172.9895	-1.75	3.85×10^{-4}	12.05
5.5164	257.9690	-1.76	2.85×10^{-4}	2.58
5.6942	245.0108	-1.76	9.47×10^{-3}	3.45
6.3325	239.0905	-1.76	3.75×10^{-2}	1.67
6.2017	217.1071	-1.77	3.82×10^{-4}	1.79
5.5168	240.9799	-1.79	8.70×10^{-4}	2.62
10.3612	253.1799	-1.81	4.87×10^{-3}	3.78
1.5198	236.9687	-1.85	8.63×10^{-3}	1.83
7.2685	270.0390	-1.86	5.98×10^{-4}	1.34
6.7945	349.0008	-1.87	3.84×10^{-5}	1.99
7.9353	330.1388	-1.88	8.67×10^{-3}	1.19
5.8297	348.0841	-1.89	3.93×10^{-4}	1.92

1.5062	251.0134	-1.89	5.98×10^{-4}	2.71
5.8977	290.0141	-1.90	2.08×10^{-3}	2.54
5.6870	334.9836	-1.92	3.86×10^{-4}	1.13
9.9938	279.2306	-1.92	4.38×10^{-3}	2.02
10.3692	321.1693	-1.92	3.86×10^{-2}	1.51
5.9021	307.0038	-1.92	3.92×10^{-2}	1.50
6.3240	171.0993	-1.93	7.04×10^{-3}	1.11
0.8802	233.0758	-1.93	8.69×10^{-4}	1.23
5.8966	222.0261	-1.98	3.07×10^{-3}	3.50
8.3415	185.1168	-1.99	3.86×10^{-3}	3.43
5.3080	261.0095	-2.01	5.67×10^{-3}	1.85
5.9032	92.9851	-2.06	8.67×10^{-3}	1.47
6.3623	363.0186	-2.08	9.64×10^{-4}	1.27
5.9452	307.0110	-2.09	3.86×10^{-2}	1.46
1.4926	363.0281	-2.10	5.90×10^{-3}	2.09
4.9512	309.1102	-2.12	4.76×10^{-4}	1.98
8.5226	439.1946	-2.13	3.82×10^{-4}	2.54
7.9082	343.1745	-2.20	6.08×10^{-3}	1.51
8.5226	371.2061	-2.21	4.97×10^{-3}	5.16
7.0010	253.0512	-2.22	8.97×10^{-4}	1.45
5.0617	331.0924	-2.23	6.85×10^{-4}	1.85
4.9479	331.0908	-2.25	3.66×10^{-3}	1.26
6.2000	179.0345	-2.29	8.79×10^{-4}	1.87
7.1130	257.1759	-2.34	6.53×10^{-4}	2.15
5.8845	290.0220	-2.35	1.94×10^{-2}	2.83
5.6461	273.0088	-2.35	6.87×10^{-3}	1.70
5.6416	203.0008	-2.35	3.84×10^{-4}	1.49
9.9464	253.1786	-2.37	2.96×10^{-3}	1.28
9.2170	589.1862	-2.42	5.97×10^{-4}	1.84
8.3433	253.1325	-2.42	3.89×10^{-3}	1.34
5.6162	269.0680	-2.44	8.67×10^{-4}	2.36
9.2177	470.2008	-2.47	1.98×10^{-3}	1.40
7.9566	226.1453	-2.48	4.79×10^{-4}	2.08
8.5246	456.1821	-2.50	5.57×10^{-4}	1.17
7.9721	556.3265	-2.50	3.85×10^{-4}	5.26
8.5224	507.1806	-2.62	6.06×10^{-5}	1.63
9.2142	385.2214	-2.62	2.74×10^{-3}	8.55
7.9609	294.1313	-2.65	7.59×10^{-3}	1.12
9.2152	453.2106	-2.68	4.76×10^{-2}	4.45
5.4352	188.0357	-2.74	5.96×10^{-3}	1.07
5.9034	425.9876	-2.75	5.32×10^{-3}	1.04
6.1924	247.0220	-2.75	5.87×10^{-4}	1.27
6.5994	337.0364	-2.81	5.84×10^{-3}	1.52
7.9704	624.3160	-2.86	3.86×10^{-3}	2.21
7.9482	226.1383	-2.90	4.87×10^{-4}	2.09

9.2177	521.1983	-2.90	6.57×10^{-3}	2.52
9.6894	354.1750	-3.00	9.47×10^{-3}	1.01
5.5826	408.0888	-3.54	5.86×10^{-3}	1.56
7.9738	692.2986	-3.58	3.86×10^{-2}	1.24
0.8226	806.4070	-5.11	9.78×10^{-4}	1.61

a) Fold change was calculated from the arithmetic mean values of the Tph2-/- mice and the control mice. Fold change with a positive value indicates a relatively higher concentration in the Tph2-/- mice, while a negative value indicates a relatively lower concentration compared with the control mice.

b) *P*-values were determined by the Mann-Whitney *U* test.

c) VIP denotes variable importance for projection where values larger than 1.00 reflects high contribution to the distinction between the Tph2-/- group and the control group.

Supplementary Table S5 Summary of the Metabolic Pathway Analysis (MetPA) results in Tph2-/- mice

No.	Pathway Name	Hits[a]	p[b]	-log(p)	Impact[c]
1	Phenylalanine, tyrosine and tryptophan biosynthesis	2	6.203E-4	7.3853	1.0
2	Phenylalanine metabolism	2	0.005447	5.2127	0.40741
3	Citrate cycle (TCA cycle)	3	9.833E-4	6.9246	0.15106
4	Purine metabolism	4	0.0044376	5.4176	0.03436
5	Tryptophan metabolism	2	0.064714	2.7378	0.10987
6	Butanoate metabolism	2	0.021388	3.8449	0.0
7	Alanine, aspartate and glutamate metabolism	2	0.025244	3.6792	0.0
8	Ubiquinone and other terpenoid-quinone biosynthesis	1	0.031444	3.4595	0.0
9	Glycine, serine and threonine metabolism	2	0.040752	3.2003	0.0
10	Glyoxylate and dicarboxylate metabolism	1	0.17529	1.7413	0.25806
11	Pyruvate metabolism	1	0.21863	1.5204	0.18375
12	Tyrosine metabolism	1	0.37846	0.97166	0.14045
13	Glycolysis or Gluconeogenesis	1	0.2436	1.4122	0.09891
14	Cysteine and methionine metabolism	1	0.25175	1.3793	0.02292
15	Arginine and proline metabolism	1	0.37846	0.97166	0.01198
16	Valine, leucine and isoleucine biosynthesis	1	0.11084	2.1996	0.0
17	Aminoacyl-tRNA biosynthesis	2	0.16334	1.8119	0.0
18	Propanoate metabolism	1	0.19289	1.6457	0.0

a) The ‘Hits’ is the number of matched compound from the uploaded data in the pathway.

b) The ‘*p*’ is the original *p* value calculated from the pathway enrichment analysis.

c) The ‘Impact’ is the pathway impact value calculated from the pathway topological analysis.

Supplementary Table S6 Perturbed metabolic pathways and associated metabolites in Tph2-/- mice

Pathway category	Pathway[a]	Associated metabolites[b]
Amino acid metabolism	Phenylalanine, tyrosine and tryptophan biosynthesis	Phenylalanine↑, Tyrosine↓
	Phenylalanine metabolism	Phenylalanine↑, Tyrosine↓, Hippurate↑
	Tryptophan metabolism	5-Hydroxyindoleacetate↓, Kynurenine↑, 3-Hydroxykynurenine↑, Xanthurenate↑
Energy metabolism	Citrate cycle	Citrate↓, Succinate↓, Pyruvate↓
	-	Creatine↓
Nucleotide metabolism	Purine metabolism	Xanthosine↓, Uric acid↓, Guanosine↑, Hypoxanthine↑
Lipid metabolism	-	LysoPC (20:4)↑, LysoPC (22:4)↑

a) The column 'Pathway' refers to the KEGG database-based pathway correlation results by MetPA.

b) '↑' indicates increased metabolite level of the Tph2-/- mice, while '↓' indicates decreased metabolite level compared with the control mice.

Supplementary Table S7 Summary of the Metabolic Pathway Analysis (MetPA) results of serotonin deficiency

No.	Pathway Name	Hits[a]	*p*[b]	-log(*p*)	Impact[c]
1	Phenylalanine, tyrosine and tryptophan biosynthesis	2	0.0011171	6.797	1.0
2	Phenylalanine metabolism	2	0.0096493	4.6409	0.40741
3	Citrate cycle (TCA cycle)	4	1.2129E-4	9.0173	0.21905
4	Tryptophan metabolism	3	0.017032	4.0727	0.19371
5	Purine metabolism	5	0.001941	6.2446	0.06726
6	Alanine, aspartate and glutamate metabolism	3	0.0040319	5.5135	0.06329
7	Butanoate metabolism	3	0.0031238	5.7687	0.0
8	Glyoxylate and dicarboxylate metabolism	1	0.22696	1.483	0.25806
9	Pyruvate metabolism	1	0.28074	1.2703	0.18375
10	Tyrosine metabolism	1	0.47018	0.75464	0.14045
11	Glycolysis or Gluconeogenesis	1	0.31127	1.1671	0.09891
12	Ubiquinone and other terpenoid-quinone biosynthesis	1	0.041777	3.1754	0.0
13	Glycine, serine and threonine metabolism	2	0.06893	2.6747	0.0
14	Valine, leucine and isoleucine biosynthesis	1	0.14523	1.9294	0.0
15	Aminoacyl-tRNA biosynthesis	2	0.25411	1.37	0.0
16	Cysteine and methionine metabolism	1	0.32118	1.1358	0.02292
17	Arginine and proline metabolism	1	0.47018	0.75464	0.01198

a) The ‘Hits’ is the number of matched compound from the uploaded data in the pathway.

b) The ‘*p*’ is the original *p* value calculated from the pathway enrichment analysis.

c) The ‘Impact’ is the pathway impact value calculated from the pathway topological analysis.

Supplementary Table S8 Perturbed metabolic pathways and associated metabolites of serotonin deficiency

Pathway category	Pathway[a]	Associated metabolites[b]
Amino acid metabolism	Phenylalanine, tyrosine and tryptophan biosynthesis	Phenylalanine↑, Tyrosine↓
	Phenylalanine metabolism	Phenylalanine↑, Hippurate↑, Tyrosine↓
	Tryptophan metabolism	Serotonin↓, 5-Hydroxyindoleacetate↓, Kynurenine↑, Kynurenate↑, 3-Hydroxykynurenine↑, Xanthurenate↑
Energy metabolism	Citrate cycle	Citrate↓, Oxoglutarate↓, Succinate↓, Pyruvate↓
	-	Creatinine↓, Creatine↓
Nucleotide metabolism	Purine metabolism	Xanthine↓, Xanthosine↓, Uric acid↓, Guanosine↑, Hypoxanthine↑
Lipid metabolism	-	LysoPC (18:4)↑, LysoPC (20:4)↑, LysoPC (22:4)↑, LysoPC (22:6)↑
Gut microflora metabolism	-	Hippurate↑, 3-Indolepropionic acid↓, Indoxyl sulfate↓

a) The column 'Pathway' refers to the KEGG database-based pathway correlation results by MetPA.

b) '↑' indicates increased metabolite level of the serotonin deficient mice, while '↓' indicates decreased metabolite level compared with the control mice.

Supplementary Table S9 The stability and repeatability of the UPLC-MS method validation in the positive (ESI+) and negative (ESI-) ion mode

Stability of the UPLC-MS method

ESI+					ESI-				
No.	Retention time/min	RSD% (n=9)	*m/z*	RSD% (n=9)	No.	Retention time/min	RSD% (n=9)	*m/z*	RSD% (n=9)
1	0.9628	0.32	657.1317	0.00025	1	1.1629	0.44	391.1207	0.00027
2	5.6324	0.10	154.0418	0.00052	2	3.0126	0.22	202.9704	0.00028
3	7.3625	0.062	324.0547	0.00042	3	6.8484	0.11	154.4972	0.00043
4	10.4732	0.082	862.5690	0.000097	4	9.0752	0.087	589.1886	0.00021
5	14.4975	0.049	279.1662	0.00037	5	11.5190	0.052	616.2779	0.00012
6	16.8164	0.054	369.3521	0.00035	6	15.4900	0.043	201.1137	0.00032

Repeatability of the UPLC-MS method

ESI+					ESI-				
No.	Retention time/min	RSD% (n=6)	m/z	RSD% (n=6)	No.	Retention time/min	RSD% (n=6)	m/z	RSD% (n=6)
1	0.9628	0.21	657.1317	0.00017	1	1.1629	0.28	391.1207	0.00025
2	5.6324	0.072	154.0418	0.00038	2	3.0126	0.17	202.9704	0.00054
3	7.3625	0.058	324.0547	0.00030	3	6.8484	0.085	154.4972	0.00033
4	10.4732	0.042	862.5690	0.00015	4	9.0752	0.081	589.1886	0.00017
5	14.4975	0.033	279.1662	0.00035	5	11.5190	0.031	616.2779	0.00020
6	16.8164	0.032	369.3521	0.00040	6	15.4900	0.042	201.1137	0.00026

QC samples were evaluated every 10 serum samples to assess system stability, while repeatability was evaluated by the six replicate QC samples. Six metabolites with different m/z values and polarities were selected for quality control/quality assurance purposes under ESI+ and ESI- modes separately.

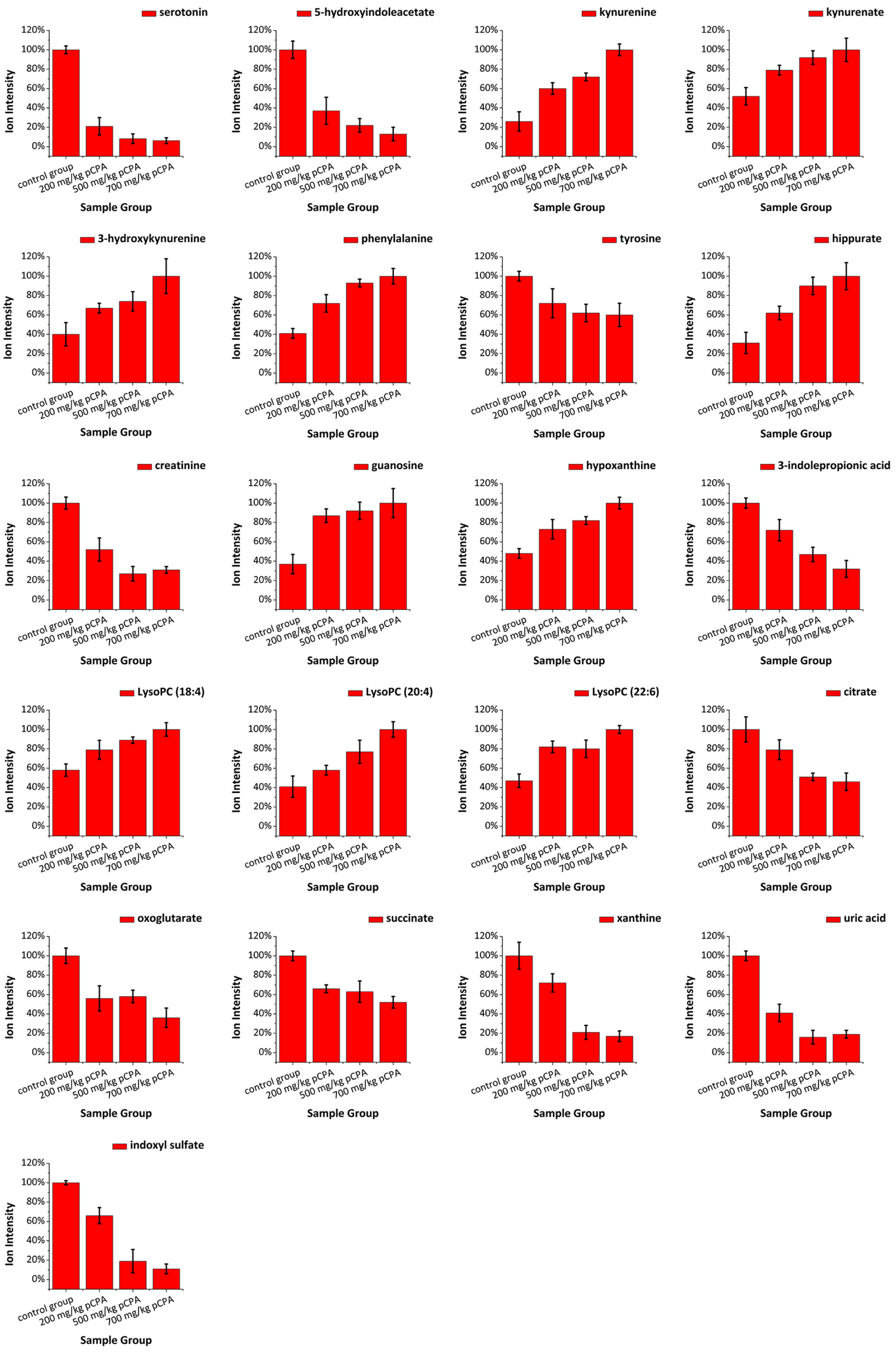

Supplementary Figure S1 Normalized mean peak areas of metabolites in mice injected with increasing dosages of pCPA compared with the control mice (n=10).

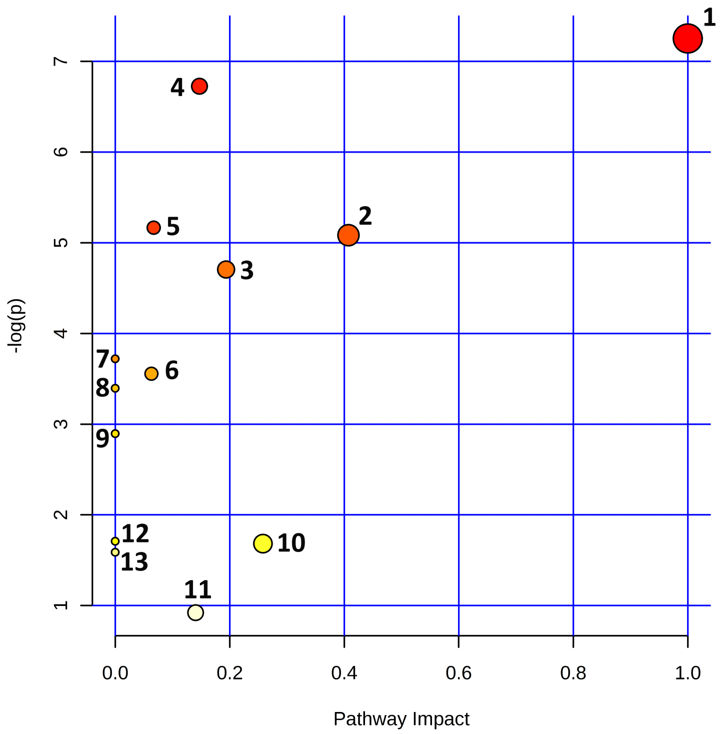

Supplementary Figure S2 Pathway analysis of the serotonin-deficiency-associated biomarkers induced by pCPA. The dots mean the serotonin-deficiency-affected pathways, with each number corresponding to the number listed in Supplementary Table S2. Pathways were arranged according to enrichment analysis (y axis) and topology analysis (x axis) scores (see "Materials and methods" section and Supplementary Table S2 for details).

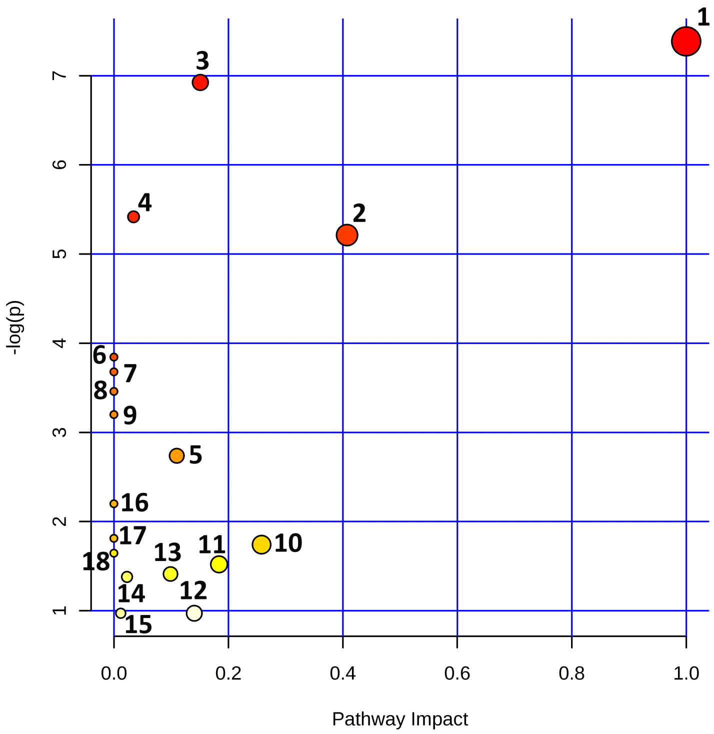

Supplementary Figure S3 Pathway analysis of the serotonin-deficiency-associated biomarkers in Tph2-/- mice. The dots mean the serotonin-deficiency-affected pathways, with each number corresponding to the number listed in Supplementary Table S5. Pathways were arranged according to enrichment analysis (y axis) and topology analysis (x axis) scores (see "Materials and methods" section and Supplementary Table S5 for details).

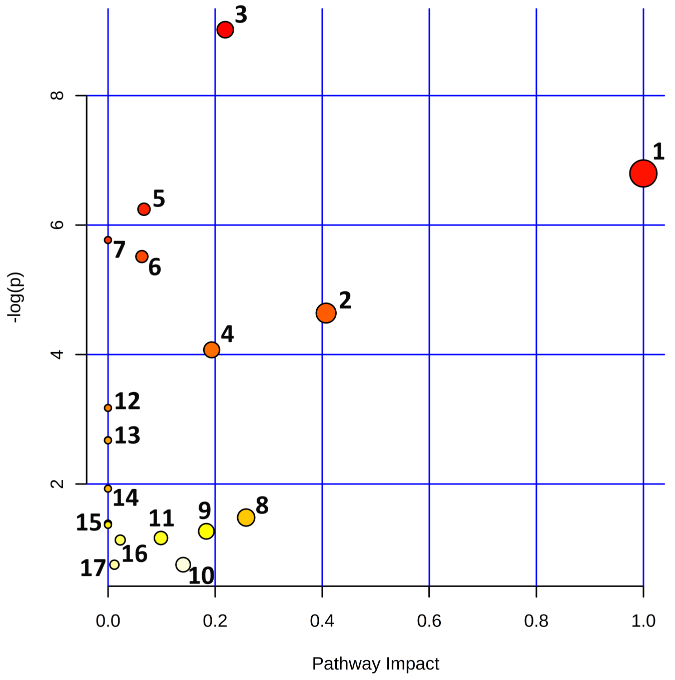

Supplementary Figure S4 Pathway analysis of the serotonin-deficiency-associated biomarkers using MetPA. The dots mean the serotonin-deficiency affected pathways, with each number corresponding to the number listed in Supplementary Table S7. Pathways were arranged according to enrichment analysis (y axis) and topology analysis (x axis) scores (see "Materials and methods" section and Supplementary Table S7 for details).
